# Adherence to the WCRF/AICR 2018 recommendations for cancer prevention and risk of cancer: prospective cohort studies of men and women

**DOI:** 10.1038/s41416-020-0806-x

**Published:** 2020-03-25

**Authors:** Joanna Kaluza, Holly R. Harris, Niclas Håkansson, Alicja Wolk

**Affiliations:** 1 Unit of Cardiovascular and Nutritional Epidemiology, Institute of Environmental Medicine, Karolinska Institutet, Stockholm, Sweden; 2 Department of Human Nutrition, Warsaw University of Life Sciences–SGGW, Warsaw, Poland; 3 Program in Epidemiology, Division of Public Health Sciences, Fred Hutchinson Cancer Research Center, Seattle, WA USA; 4 Department of Epidemiology, University of Washington, Seattle, WA USA; 5 Department of Surgical Sciences, Uppsala University, Uppsala, Sweden

**Keywords:** Risk factors, Cancer epidemiology

## Abstract

**Background:**

In 2018, the World Cancer Research Fund/American Institute for Cancer Research (WCRF/AICR) issued revised recommendations for cancer prevention. We examined the relation between adherence to these recommendations and risk of total cancer in two population-based Swedish prospective cohorts (29,451 men and 25,349 women).

**Methods:**

Standardized-WCRF/AICR 2018 and simplified-WCRF/AICR 2018 adherence scores were constructed based on the WCRF/AICR recommendations for body weight, physical activity, diet, alcohol consumption and dietary supplement use. Data were collected using a self-administered questionnaire.

**Results:**

During the 15.4 years of follow-up, 12,693 incident cancers were ascertained. The multivariable HR between extreme categories of the Standardized-WCRF/AICR 2018 score (4.1–7 vs. 0–2) was 0.88 (95% CI = 0.82–0.95) and for the Simplified score (5–8 vs. 0–2) was 0.85 (95% CI = 0.80–0.90); each 1-score increment in recommendation adherence was associated with 3% (95% CI = 1–5%) and 4% (95% CI = 2–5%) decreased risk, respectively. Based on the Simplified scoring, most participants (>90%) did not meet WCRF/AICR 2018 recommendations regarding consumption of plant foods, limited consumption of red/processed meat and ‘fast food’/processed food, and <50% of participants met the weight and physical activity recommendations.

**Conclusions:**

Adherence to the 2018WCRF/AICR recommendations substantially reduced the risk of total cancer. Given that many people do not meet the recommendations, there is a great potential for cancer prevention.

## Background

In 2018, the World Cancer Research Fund (WCRF) and the American Institute for Cancer Research (AICR) issued revised recommendations for cancer prevention (WCRF/AICR 2018) to reduce the global burden of cancer.^1^ In the WCRF/AICR 2018 Expert Report as compared with the previous WCRF/AICR 2007 report, some recommendations were reformulated or replaced by others. In particular, the recommendation to “limit consumption of energy-dense foods; avoid sugary drinks” was replaced by two recommendations: (1) “limit consumption of ‘fast food’ and other processed food high in fat, starches or sugar” and (2) “limit consumption of sugar sweetened drinks”. Further, the recommendation to “limit consumption of salt; avoid mouldy cereals (grains) or pulses (legumes)” was removed.^1,2^ The recommendation to “eat a diet rich in whole grains, vegetables, fruit and beans” was also added, and it was clearly stated to consume a diet that provides at least 30 g/day of fibre from food sources.^1^

In 2019, a standardised scoring system was developed to examine the adherence to the WCRF/AICR 2018 Recommendations in relation to cancer risk in populations.^3^ The Standardized-WCRF/AICR 2018 score was created in collaboration between researchers at the U.S. National Cancer Institute (NCI) and members of AICR and WCRF International, and in consultation with the WCRF/AICR Expert Panel and other researchers.^3^ In addition to assigning a full point for meeting a recommendation, the Standardized-2018 score gives points for partial adherence. We had simultaneously developed our own score (Simplified-WCRF/AICR 2018 score) to evaluate the WCRF/AICR recommendations, with a scoring system that gave one point for meeting a recommendation, and zero points for not meeting the recommendation, with no points for partial adherence.

In this study, we prospectively evaluated the association between adherence to the WCRF/AICR 2018 recommendations and total cancer risk, using both the Standardized-WCRF/AICR 2018 score and the Simplified-WCRF/AICR 2018 score in two large cohorts of middle-aged and elderly men and women.

## Methods

### Study population

Established in 1997, the Cohort of Swedish Men (COSM) included men, born 1918–1952, who lived in Västmanland and Örebro counties (central Sweden), and completed a questionnaire about diet and lifestyle. The Swedish Mammography Cohort (SMC) was established in 1987, when all women, born 1914–1948, from Västmanland and Uppsala counties, were invited to participate in a mammography screening programme. In 1997, women from the SMC completed the same questionnaire as the COSM participants, except for some sex-specific questions. In late autumn 1997, the questionnaire was returned by 48,850 men and 39,227 women. Participants of both cohorts well represented the general Swedish population in terms of age distribution, level of education as well as prevalence of obesity.^4^

**Fig. 1:**
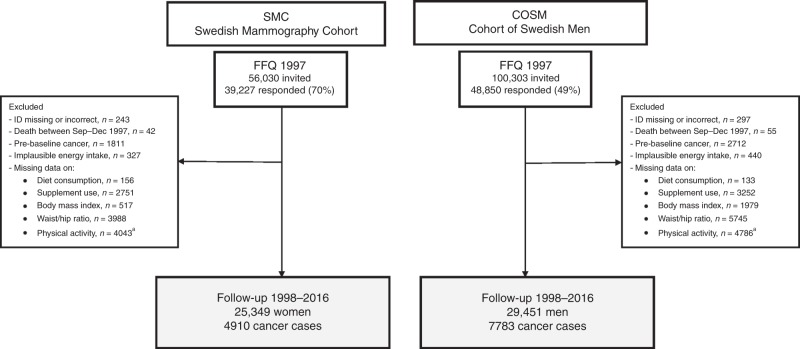
Flow chart of the Swedish Mammography Cohort (SMC) and the Cohort of Swedish Men (COSM). FFQ, food-frequency questionnaire. ^a^Missing data on heavy manual labour or time spent on exercise or walking/cycling.

A flow chart detailing the analytic study population is shown in Fig. 1. We excluded participants with an incorrect or missing personal identification number (297 men and 243 women), those who died prior to the start of follow-up, 1 January 1998 (55 men and 43 women), or those with a previous cancer other than non-melanoma skin cancer (2712 men and 1810 women). Moreover, the participants with extreme energy intake (440 men and 327 women; ±3 SDs from the mean value for log_e_-transformed estimates calculated separately for men and women), and those with missing data on diet (133 men and 156 women), dietary supplement use (3525 men and 2751 women), body mass index (BMI) (1979 men and 517 women), waist-to-hip ratio (5745 men and 3988 women) or physical activity (4786 men and 4043 women) were excluded. Finally, 29,451 men and 25,349 women remained for the analysis.

### Data collection

Information on education level, smoking status, weight, height, physical activity, diet, use of dietary supplements and medication use, including aspirin use, was collected on the questionnaire in 1997. BMI was calculated by dividing body weight (kilograms) by height squared (meters). Questions about physical activity were validated using 7-day activity reports and accelerometers among 116 participants, 56–75 years old, and correlations between total daily activity estimated by the questionnaire and measured by the accelerometers and by the 7-day activity reports were 0.38 (95% CI: 0.22–0.54) and 0.64 (95% CI: 0.45–0.83), respectively.^5^

Food and alcohol consumption was assessed with a validated 96-item food-frequency questionnaire (FFQ).^6^ Participants were asked to indicate how often over the previous year they had consumed each item by using eight predefined frequency categories, ranging from “never” to “≥3 times per day”. The frequencies of food consumption were converted to gram per day by multiplying the frequency of consumption of each food item by an appropriate age-specific portion size.

The questionnaire also included questions on family history of cancer and history of diabetes. Moreover, information on diagnosis of diabetes was collected via linkage with the Swedish National Diabetes Register and the Swedish National Patient Register (ICD-10 codes: E10–E14). Sex-specific information, such as hormone replacement therapy use, parity and age at first birth, was collected in the SMC.

### Standardized- and Simplified-WCRF/AICR 2018 scoring criteria

The Standardized-WCRF/AICR 2018 and the Simplified-WCRF/AICR 2018 scores were calculated according to scoring criteria presented in Table 1. The components and scoring criteria to calculate the Standardized-WCRF/AICR 2018 score were based on the previously published guidelines by Shams-White et al.^3^ For the Standardized-WCRF/AICR 2018 score, 1 point is assigned for recommendation adherence, 0.5 points are assigned for partially following the recommendations and 0 points are assigned for those furthest from meeting the recommendations (scores 0, 0.5 and 1). For recommendations that included sub-recommendations (e.g. BMI and waist circumference as two parts of the ‘healthy weight’ recommendation), each sub-recommendation was scored separately with points of 0.5 given for fully meeting the sub-recommendation, 0.25 for partially meeting the sub-recommendation and 0 for those furthest from meeting the sub-recommendation, with the full recommendation still scoring a maximum of 1.0 when both sub-recommendations were met. In the Simplified-WCRF/AICR 2018 score, developed by our group, 1 point is assigned when fully meeting the criteria, and 0 when the criteria were not met (scores 0 or 1). After summing the points for each recommendation, the Standardized-WCRF/AICR 2018 score ranges from 0 to 7, and the Simplified-WCRF/AICR 2018 score ranges from 0 to 8, due to inclusion of the non-use of dietary supplements to the Simplified score but not to the Standardized score.

**Table 1:** WCRF/AICR 2018 recommendations for cancer prevention—scoring components and criteria.

Standardized-WCRF/AICR 2018 score^3^		Simplified-WCRF/AICR 2018 score	
Components	Scoring criteria	Components	Scoring criteria
*Recommendation 1—be a healthy weight*			
BMI (kg/m^2^)		BMI (kg/m^2^)	
18.5–24.9	0.5	18.5–24.9^a^	1
25–29.9	0.25		
<18.5 or ≥30	0		
Waist circumference (cm)			
Men/ Women			
<94/< 80	0.5		
94–101.9/80–87.9	0.25		
≥102/≥88	0		
*Recommendation 2—be physically active*			
Moderate–vigorous physical activity (min/wk)			
≥150	1	Heavy manual labor *or* walking/cycling ≥40 min/day *or* exercise ≥4 h/week^b^	1
75–149.9	0.5		
<75	0		
*Recommendation 3—eat a diet rich in whole grains, vegetables, fruits and beans*			
Fruits and vegetables (g/day)			
≥400	0.5	Whole grains ≥175 g/day^c^ and non-starchy vegetables/fruits/beans ≥400 g/day^d^ and dietary fibre ≥30 g/day^d^	1
200–399.9	0.25		
<200	0		
Total fibrer (g/day)			
≥30	0.5		
15–29.9	0.25		
<15	0		
*Recommendation 4—Limit the consumption of ‘fast foods’ and others processed high in fat, starches or sugars*			
Tertile 1	1	<2 Servings/day^e^	1
Tertile 2	0.5		
Tertile 3	0		
*Recommendation 5—Limit the consumption of red and processed meat*			
Red meat <500 and processed meat <21 g/week	1	Red meat <500 g/week^f^ and processed meat 0 g/week^f^	1
Red meat <500 and processed meat 21–99.9 g/week	0.5		
Red meat >500 or processed meat ≥100 g/week	0		
*Recommendation 6—limit the consumption of sugar-sweetened drinks*			
0 g/day	1	<1 Serving/day^g^	1
0.1–250 g/day	0.5		
≥250 g/day	0		
*Recommendation 7—Limit alcohol consumption*			
0 drinks/day	1	≤2 drinks/day men; ≤1 drink/day women^h^	1
≤2 drinks/day men; ≤1 drink/day women	0.5		
>2 drinks/day men; >1 drink/day women	0		
*Recommendation 8—do not use supplements for cancer prevention*			
Not included in the score		Not using supplements *or* using non-regular^i^	1

*BMI* body mass index, *WCRF/AICR* World Cancer research Fund/American Institute for Cancer Research, *n/s* no scoring.

^a^World Health Organization’s classification of normal weight.^18^

^b^Scoring cut-offs were determined empirically, including the previously published result of the study conducted in the SMC and the COSM.^19,20^

^c^Scoring cut-off was determined empirically; whole grains included granary/whole-meal bread, crispbread, bran wheat/oats, cereals/muesli and dry oatmeal porridge and other porridge/gruel.

^d^Scoring cut-offs according to the WCRF/AICR 2018 recommendations:^1^ non-starchy vegetables/fruits included lettuce, spinach, cabbage, cauliflower, broccoli/Brussels sprouts, carrots, beetroots, peppers, tomatoes, onion/leak, garlic, green peas and mixed frozen vegetables, apples/pears, bananas, orange/citrus fruits, fresh/frozen berries and other fruits; beans included one question about beans/lentils/pea soup consumption.

^e^Scoring cut-off was determined empirically; ‘Fast foods’ and others processed high in fat, starch or sugar products were classified based on the NOVA classification system,^21^ and included sugar/honey, white bread, pizza, fried potatoes, French fries, chips/popcorn/cheese puffs, buns/cakes, biscuits/wafers/rusks, gateau/pastries, sweets, chocolate, ice cream and margarines/spreads, full-fat salad dressing, full-fat mayonnaise, full-fat crème fraiche and full-fat cream.

^f^Scoring cut-offs according to the WCRF/AICR 2018 recommendations:^1^ red meat (unprocessed) included pork, beef/veal and minced meat; processed meat included sausages, cold cuts/ham/salami, blood pudding/sausages and liver paté.

^g^Scoring cut-off was determined empirically; sugar-sweetened drinks included soft drinks/soda and juices.

^h^In all, 12 g of ethanol corresponds to one drink; alcohol included class I beer (<2.25% ethanol by volume), class II beer (2.80–3.50%), class III beer (>3.50%), wine, strong wine (>18% alcohol) and liquor.

^i^Scoring cut-off based on the WCRF/AICR 2018 recommendations.^1^

### Identification of cancer cases and follow-up

Incident cases of total cancer were identified by linkage to the National Cancer Register, which is nearly 100% complete.^7^ Cancer cases were classified according to the International Classification of Diseases and Related Health Problems, 10th Revision (codes: C00-D48). Participants were followed from January 1, 1998, to the date of cancer diagnosis, death or the end of follow-up (December 31, 2016), whichever occurred first.

### Statistical analysis

Cox proportional hazard regression models were used to estimate the hazard ratios (HRs) and 95% confidence intervals (CIs) of the risk of total cancer in the combined cohorts, and separately for men and for women. Both scores, the Standardized and the Simplified-WCRF/AICR 2018 scores were divided into three categories of adherence: (1) low—0–2 points (reference group), (2) medium—2.1–4 (Standardized score), 3–4 points (Simplified score) and (3) high—4.1–7 points (Standardized score), 5–8 points (Simplified score).

The multivariable HRs were adjusted for age of participants in 1998 (years, continuous), sex, education (less than high school, high school and university), smoking status and pack-years of smoking (never; ex-smokers <20, 20–39 and ≥40 pack-years; current <20, 20–39 or ≥40 pack-years), height (centimetres and quartiles), aspirin use (yes, no), history of diabetes (yes, no) and family history of cancer (yes, no). In addition, the multivariable HRs for women were adjusted for hormone replacement therapy use (ever, never) and parity/age at first birth (nulliparous, age at first birth <26/1–2 children, age at first birth <26/≥3 children, age at first birth 26–30/1–2 children, age at first child birth 26–30/≥31 years, age at first birth ≥31/1–2 children and age at first birth ≥31/≥3 children). Moreover, due to the lack of inclusion of dietary supplement use in the Standardized score by its creators,^3^ we adjusted the multivariable HRs for this score for supplement use (regular, no/non-regular). Missing data on educational level (0.4%), smoking status (1.2%), aspirin use (8.7%), hormone replacement therapy use (2.6%), parity (2.2%) and age at first birth (10.6%) were included in the models as separate categories.

Secondary analysis was conducted by examining associations between meeting (yes, no) each individual WCRF/AICR 2018 recommendation and the risk of total cancer using the Simplified scoring system, and these associations were adjusted as described above, and were mutually adjusted for the other recommendations.

The proportional hazard assumption was evaluated for both scores by regressing scaled Schoenfeld residuals against survival time, and there was no evidence of departure from the assumption. To calculate *P* values for trend, the WCRF/AICR 2018 scores were used as a continuous variable. A likelihood-ratio test was used to assess for effect modification by gender in relation to the risk of total cancer incidence. Furthermore, the shape of the associations between the Standardized- and Simplified-WCRF/AICR 2018 scores and total cancer incidence was examined by using a restricted cubic-spline regression analysis with three knots (at the 10th, 50th and 90th percentile).^8^ Moreover, sensitivity analyses were conducted by excluding the first 3 years of follow-up, and excluding participants who were diagnosed with diabetes before baseline.

Statistical analyses were performed using Stata 14.2 (StataCorp, College Station, TX); two-sided *P* values ≤ 0.05 were recognised statistically significant.

## Results

The median (range) of the Standardized-WCRF/AICR 2018 adherence score was 3.25 points (0–7), and the Simplified-WCRF/AICR 2018 score was 3.0 points (0–8). The Spearman correlation coefficient between both scores was 0.60 (*P* value < 0.001). Age-standardised baseline characteristics of participants by categories of the Standardized- and Simplified-WCRF/AICR 2018 scores are presented in Table 2. Men were less likely to fall into the highest score category for the Standardized, but this pattern was not observed to the same extent for the Simplified score. Compared with participants in the lowest categories of both scores, a lower percentage of those in the highest score categories had hypertension and were current or ex-smokers. With increasing Standardized and Simplified scores, mean BMI decreased, and physical activity increased. As expected, with higher values of both scores, consumption of whole grains, fruits, vegetables and beans increased, while consumption of ‘fast foods’ and other foods high in fat, starches and sugars, as well as sugar-sweetened drinks, unprocessed and processed red meat and alcohol decreased. The percentage of participants who did not use dietary supplements or who did not use supplements regularly decreased as the Standardized score increased, while for the Simplified score, the opposite participant distribution was observed.

**Table 2:** Age-standardised baseline characteristics of 54,800 Swedish men and women by categories of the Standardized- and the Simplified-World Cancer Research Fund/American Institute for Cancer Research 2018 recommendation score (Standardized- and Simplified-WCRF/AICR 2018 score).

Characteristics	Standardized-WCRF/AICR 2018 score, range (median)			Simplified-WCRF/AICR 2018 score, range (median)		
	0–2 (1.75)	2.1–4 (3.25)	4.1–7 (4.50)	0–2 (2)	3–4 (3)	5–8 (5)
Number of people	5,212	40,161	9,427	10,727	34,961	9,112
Men, %	68.9	55.0	40.1	56.7	52.9	53.4
Age at baseline, years	59.5 ± 9.3	60.6 ± 9.2	61.9 ± 9.0	60.4 ± 9.2	60.8 ± 9.2	61.0 ± 9.1
University education, %	12.7	17.9	23.6	18.9	17.9	19.2
Smoking status, %						
Never	33.2	44.5	51.2	39.8	45.2	48.0
Ex-smokers	37.5	33.3	29.3	35.6	31.7	30.2
Current smokers	28.6	22.2	18.0	23.6	22.1	20.4
Aspirin use, %	42.2	39.4	36.7	43.6	38.9	35.2
Hypertension, %	30.8	22.9	18.2	28.2	22.5	17.6
Diabetes, %	7.5	6.4	7.0	7.0	6.5	6.8
Family history of cancer, %	33.9	33.8	32.9	34.5	33.4	33.6
Height, cm	174 ± 9	172 ± 9	170 ± 9	172 ± 9	171 ± 9	172 ± 9
Energy intake, kcal/day	2573 ± 886	2290 ± 835	1986 ± 705	2377 ± 837	2237 ± 823	2245 ± 866
Nulliparous, %^a^	6.2	7.8	9.7	8.3	8.0	8.4
Age at first birth, years^a^	23.4 ± 4.9	24.0 ± 4.8	24.1 ± 4.9	23.9 ± 4.7	24.0 ± 4.9	24.2 ± 4.7
Hormone replacement therapy use, %^a^	41.5	46.6	46.7	49.2	46.1	44.0
*WCRF/AICR 2018 score components*						
BMI, kg/m^2^	28.2 ± 4.0	25.4 ± 3.5	23.7 ± 2.8	27.1 ± 3.6	25.3 ± 3.6	23.4 ± 2.4
Heavy manual labor, %	2.0	4.1	3.9	1.2	4.0	6.5
Walking/cycling ≥40 min/day, %	9.3	32.1	58.7	9.9	33.4	68.0
Exercise ≥4 h/week, %	5.7	23.4	46.5	7.4	25.0	50.3
Whole grains, gram/day	123 ± 99	142 ± 97	150 ± 97	128 ± 91	138 ± 95	170 ± 110
Fruit/vegetables/beans, gram/day	257 ± 148	355 ± 198	472 ± 251	344 ± 199	357 ± 207	422 ± 234
Dietary fibre intake, gram/day	25.3 ± 11.3	27.51 ± 11.5	29.1 ± 11.8	26.3 ± 10.4	27.1 ± 11.2	30.9 ± 13.5
Fast food/other food high in fat/ starches/sugar, servings/day	10.6 ± 4.7	7.6 ± 4.6	4.4 ± 3.2	7.8 ± 4.5	7.4 ± 4.7	6.5 ± 4.8
Sugar-sweetened drinks, servings/day	1.5 ± 1.6	0.8 ± 1.1	0.3 ± 0.7	1.4 ± 1.5	0.7 ± 1.0	0.3 ± 0.6
Unprocessed red meat, gram/day	58 ± 37	52 ± 37	40 ± 36	54 ± 39	50 ± 37	47 ± 39
Processed red meat, gram/day	43 ± 27	37 ± 27	25 ± 28	39 ± 29	35 ± 27	32 ± 28
Alcohol, drinks/day^b^	1.8 ± 2.8	1.0 ± 1.4	0.6 ± 0.8	1.6 ± 2.0	0.9 ± 1.5	0.6 ± 0.9
No use/non-regular supplement use, %	85.3	80.7	72.9	57.8	82.9	93.7

^a^Results presented for women.

^b^In all, 12 g of ethanol corresponds to one drink.

**Fig. 2:**
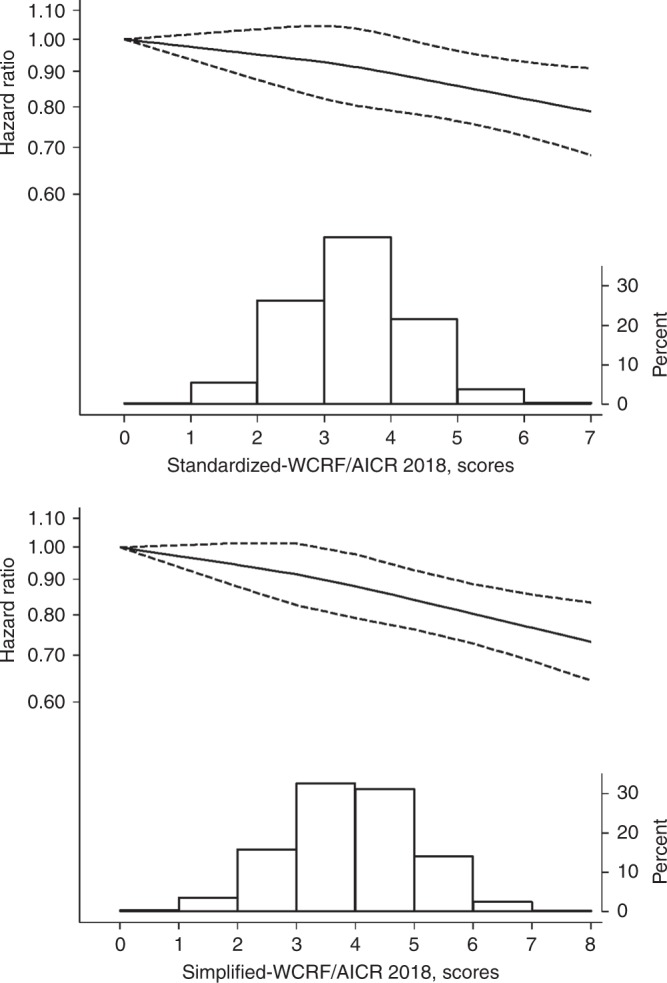
Multivariable-adjusted hazard ratio of total cancer incidence as a function of adherence of the World Cancer Research Fund/American Institute for Cancer Research (WCRF/AICR) 2018 recommendations. The solid curve shows the restricted cubic spline, and dashed–dotted lines show 95% confidence intervals. Distribution of participants according to the Standardized and Simplified-WCRF/AICR 2018 scores is presented as a histogram at the bottom of the figure.

During an average 15.4 years of follow-up (841,610 person-years; 1998–2016), 12,693 participants (7783 men and 4910 women) were diagnosed with cancer. Statistically significant associations were observed between categories of the Standardized-WCRF/AICR 2018 score and total cancer incidence in the overall study population, as well as in men and in women when examined separately (Table 3). Participants in the highest category of the Standardized-WCRF/AICR 2018 score (4.1–7) compared with those in the lowest category (0–2) had a lower risk of cancer, HR = 0.88 (95% CI = 0.82–0.95) with HRs of 0.86 (95% CI = 0.79–0.95) in men and 0.87 (95% CI = 0.77–0.99) in women. No statistically significant interaction was observed between the Standardized-WCRF/AICR 2018 score and sex for the risk of total cancer (*P* interaction = 0.52). Examining the shape of the association between risk of cancer and adherence to the WCRF/AICR 2018 recommendations using the Standardized score, we observed a linear dose–response relationship (Fig. 2); each 1-point increment was associated with a 3% (95% CI = 1–5%; *P*-trend = 0.001) lower risk of cancer.

**Table 3:** Hazard ratios (HRs) and 95% confidence intervals (CIs) of total cancer incidence by the categories of the Standardized-WCRF/AICR 2018 score and the Simplified-WCRF/AICR 2018 score in the Cohort of Swedish Men and the Swedish Mammography Cohort, follow-up 1998–2016.

	Standardized-WCRF/AICR 2018 score, range (median)			Per 1 point of WCRF/AICR	*P*-trend
	0–2 (1.75)	2.1–4 (3.25)	4.1–7 (4.50)		
Men and women *(n* = 54,800)					
Cases	1289	9353	2051		
Person-years	77,607	617,476	146,527		
Age-adjusted HR (95% CI)	1.00	0.86 (0.81–0.91)	0.74 (0.69–0.80)	0.91 (0.89–0.93)	<0.001
Multivariable-adjusted HR (95% CI)^a,b^	1.00	0.94 (0.88–0.99)	0.88 (0.82–0.95)	0.97 (0.95–0.99)	0.001
Men (*n* = 29,451)					
Cases	958	5812	1013		
Person-years	52,851	328,817	55,207		
Age-adjusted HR (95% CI)	1.00	0.88 (0.82–0.94)	0.81 (0.74–0.89)	0.95 (0.92–0.97)	<0.001
Multivariable-adjusted HR (95% CI)^a,b^	1.00	0.91 (0.85–0.97)	0.86 (0.79–0.95)	0.97 (0.94–0.99)	0.013
Women (*n* = 25,349)					
Cases	331	3541	1038		
Person-years	24,755	288,659	91,320		
Age-adjusted HR	1.00	0.92 (0.82–1.03)	0.84 (0.74–0.95)	0.94 (0.91–0.97)	<0.001
Multivariable-adjusted HR (95% CI)^a,b,c^	1.00	0.95 (0.85–1.06)	0.87 (0.77–0.99)	0.95 (0.92–0.98)	0.001
	Simplified-WCRF/AICR 2018 score, range (median)			Per 1 point of WCRF/AICR	*P*-trend
	0–2 (2)	3–4 (3)	5–8 (5)		
Men and women (*n* = 54,800)					
Cases	2613	8118	1962		
Person-years	161,674	537,718	142,218		
Age-adjusted HR (95% CI)	1.00	0.91 (0.87–0.95)	0.82 (0.77–0.87)	0.95 (0.94–0.97)	<0.001
Multivariable-adjusted HR (95% CI)^a^	1.00	0.94 (0.90–0.98)	0.85 (0.80–0.90)	0.96 (0.95–0.98)	<0.001
Men (*n* = 29,451)					
Cases	1631	4931	1221		
Person-years	89,494	274,706	72,676		
Age-adjusted HR (95% CI)	1.00	0.93 (0.88–0.98)	0.82 (0.76–0.88)	0.96 (0.94–0.98)	<0.001
Multivariable-adjusted HR (95% CI)^a^	1.00	0.94 (0.89–1.00)	0.85 (0.79–0.91)	0.97 (0.95–0.98)	0.001
Women (*n* = 25,349)					
Cases	982	3,187	741		
Person-years	72,180	263,013	69,542		
Age-adjusted HR (95% CI)	1.00	0.89 (0.83–0.96)	0.79 (0.72–0.87)	0.94 (0.91–0.96)	<0.001
Multivariable-adjusted HR (95% CI)^a,c^	1.00	0.90 (0.84–0.97)	0.80 (0.73–0.88)	0.94 (0.92–0.97)	<0.001

^a^Adjusted for age (years, continuous), sex, education (less than high school, high school or university), smoking status and pack-years of smoking (never, past <20, 20–39 or ≥40 pack-years, or current <20, 20–39 or ≥40 pack-years), height (centimetres, quartiles), history of diabetes (yes, no), aspirin use (yes, no) and family history of cancer (yes, no).

^b^Adjusted for covariates above plus dietary supplement use (regular or no/non-regular).

^c^Additionally adjusted for hormone replacement therapy use (ever, never) and parity/age at first birth (nulliparous, age at first birth <26/1–2 children, age at first birth <26/≥3 children, age at first birth 26–30/1–2 children, age at first birth 26–30/≥31 years, age at first birth ≥31/1–2 children and age at first birth ≥31/≥3 children).

The results obtained using the Simplified-WCRF/AICR 2018 score were similar to those obtained using the Standardized-WCRF/AICR 2018 score (Table 3). Participants in the highest category of the Simplified-WCRF/AICR 2018 score (5–8) compared with those in the lowest category (0–2) had lower risk of cancer, with HRs of 0.85 (95% CI = 0.80–0.90) in total participants, 0.85 (95% CI = 0.79–0.91) in men and 0.80 (95% CI = 0.73–0.88) in women. Each 1-point increment in the Simplified score was associated with a 4% (95% CI = 2–5%; *P*-trend < 0.001) lower risk of cancer in participants.

In a sensitivity analyses, we excluded the first 3 years of follow-up (excluding 1475 men and 861 women, including 934 and 631 cancer diagnoses, respectively); the HR for cancer risk between the highest versus the lowest category of the Simplified-WCRF/AICR 2018 score was comparable to the results including all participants (HR = 0.85, 95% CI = 0.80–0.92). Further exclusion of participants who were diagnosed with diabetes at pre-baseline (additional excluding 2,304 men and 1,010 women, and 585 and 178 cancer diagnoses) slightly decreased the observed associations (HR = 0.82, 95% CI = 0.77–0.88).

**Fig. 3:**
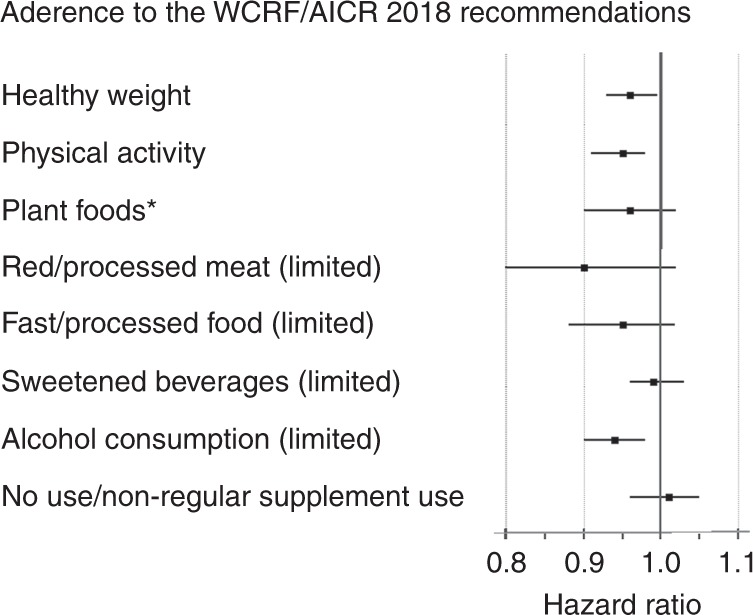
Adherence to the individual WCRF/AICR 2018 recommendations according to the Simplified scoring system in the relation to total cancer incidence in the Cohort of Swedish Men and the Swedish Mammography Cohort, follow-up 1998–2016. Results adjusted for age, sex, education, smoking status and pack-years of smoking, height, aspirin use, history of diabetes, family history of cancer and mutually adjusted for each other. * including consumption of whole grains, vegetables, fruits and beans.

We also examined the association between each individual WCRF/AICR 2018 recommendation and total cancer incidence using the Simplified score (Fig. 3). Meeting the recommendations for healthy weight and physical activity were associated with a 4% (95% CI = 0–7%) and 5% (95% CI = 2–9%), respectively, decreased risk of total cancer. Meeting the recommendations for limited red/processed meat and alcohol consumption were associated with a 10% (95% CI = −2–20%) and 6% (95% CI = 2–10%) decreased risk of cancer, respectively.

**Fig. 4:**
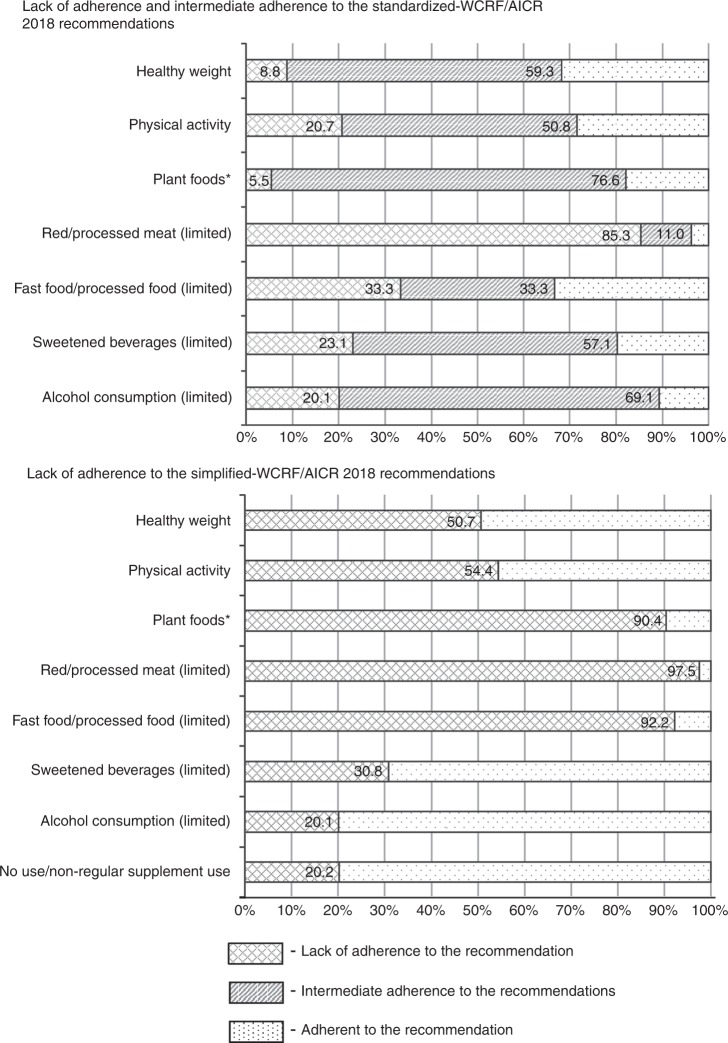
Lack of adherence, intermediate adherence (if applicable), and adherence to the individual WCRF/AICR 2018 recommendations according to the Standardized and Simplified scoring system. Percent distribution of participants meeting the individual WCRF/AICR 2018 recommendations according to both scoring systems.

The majority of the study population did not meet the specific individual recommendations. This was consistent whether the recommendations were operationalised using the Simplified or Standardized scores (Fig. 4). The recommendations least likely to be met were limiting the consumption of red and processed meat (98% of participants based on the Simplified score), limiting ‘fast food’ and other processed foods high in fat, starches or sugar (92%) and consuming plant foods, i.e. diet rich in whole grains, vegetables, fruit and beans (90%). Moreover, half of the study population did not meet the healthy weight and the physical activity criteria, 51% and 54%, respectively.

## Discussion

In these two population-based prospective cohorts of men and women, adherence to the WCRF/AICR 2018 recommendations for cancer prevention was associated with reduced risk of total cancer. Depending on the score used, each 1-point increment in adherence to the WCRF/AICR 2018 recommendations was associated with a 3–4% lower risk of cancer.

To the best of our knowledge, this is the first study examining adherence to the revised WCRF/AICR 2018 recommendations for cancer prevention in relation to total cancer incidence. Our results are in line with previous findings from prospective studies that assessed the association with the 2007 recommendations.^9–12^ Adherence to the WCRF/AICR 2007 recommendations was associated with reduced risk of total cancer^9–12^ and of some specific cancers,^9–13^ as well as reduced risk of total cancer mortality.^14–16^

The results obtained using the Simplified-WCRF/AICR 2018 score were slightly stronger than those obtained using the recently developed Standardized-WCRF/AICR 2018 score, which was designed to provide consistency when comparing WCRF/AICR recommendation adherence across studies.^3^ The Simplified score developed by our group differs from the Standardized score in that it is more rigorous, including only options of “yes” or “no” for compliance, than the Standardized score, which provides partial credit for lower levels of compliance. It should also be noted that the Simplified score may be easier to use as it does not include sub-recommendations and partial adherence; thus, it could be easier to communicate to the general public. Thus, individuals can more easily estimate their adherence with the WCRF/AICR 2018 recommendations using the Simplified score than the Standardized score.

The results obtained using the Simplified score are informative, regarding adherence to specific recommendations. Analyses of individual WCRF/AICR 2018 recommendations indicate that healthy weight, high physical activity and limited consumption of alcohol were associated with statistically significant lower risk of total cancer. Similarly, results of the European Prospective Investigation into Cancer and Nutrition (EPIC) study for the WCRF/AICR 2007 recommendations demonstrated that body fatness, physical activity and moderation in alcohol consumption were associated with total cancer incidence in participants of nine European countries.^10^ In the Alberta’s Tomorrow Project (25,100 men and women, 2,066 cancer cases), of the WCRF/AICR 2007 recommendations, only physical activity and fruit and vegetable consumption were associated with decreased cancer risk in women, but none in men.^11^

Lack of adherence to the WCRF/AICRF 2018 recommendations by a high percentage of men and women in the study population provides insight into which modifiable lifestyle areas are in most need of information and education, and strongly indicates a need for societal- level education for primary cancer prevention. The observed lack of compliance to specific recommendations provides information regarding which recommendations are critical for cancer prevention at a population level. The observed associations between specific recommendations and risk of total cancer overlap with the high percentage of population who did not meet these recommendations. Recommendations about limiting red and processed meat consumption were not met by 98%, keeping a healthy weight by 51% and being physically active by 54%. Moreover, a high percentage of the study population did not meet the recommendation to consume a diet rich in whole grains, vegetables, fruits and beans (90%), and to limit the consumption of ‘fast foods’ and other processed foods high in fat, starches or sugar (92%). Thus, increasing adherence to these specific recommendations via different means of information and education may be crucial in cancer prevention.

Our study has several strengths, including a population-based prospective design, detailed information on modifiable lifestyle factors, including diet and physical activity and a large number of incident cancer cases. Participants in the cohorts well represented the study population regarding education and body mass index.^17^ An additional strength of the study is the complete follow-up by linkage with the Swedish Cancer Register and the Swedish Cause of Death Register and the completeness of these registers. The available data on potential risk factors for cancer incidence allowed us to adjust the results for confounders; however, unmeasured or residual confounding cannot be disregarded. The FFQ had a high validity for the intake of macro- and micronutrients,^6^ and data on physical activity have also been validated;^5^ however, error in the measurement of those factors is likely, but would only result in a conservative bias of presented estimates. A limitation of our study is to not include all WCRF/AICR recommendations in the WCRF/AICR 2018 scores, i.e. “breastfeed your baby, if you can”, because there were no data available in women and it was not applicable in men. Due to these limitations, both scores, the Standardized and Simplified, may underestimate associations between adherence to the recommendations and total cancer incidence. Generalisability of our results may also be limited. In other populations, the recommendations may be met to a different degree. Therefore, the association between adherence to the recommendations and risk of cancer may differ from the results obtained in this study, and be determined by the specific study population.

In conclusion, the results of our study indicate that adherence to the WCRF/AICR 2018 recommendations is associated with decreased risk of total cancer, and a large percentage of the study population did not meet these recommendations. Our findings provide robust evidence that the guidelines for cancer prevention should be widely disseminated in society. Low degree of adherence to most of the recommendations highlights the importance of the promotion recommendations in ongoing and future cancer prevention efforts.

## Data Availability

The data and the analytical programme are stored on a highly secure institutional server under the supervision of A. Wolk (PI). Investigators may apply to access the study’s deidentified data through contact with the PI.
